# Physicians’ Use of Email With Patients: Factors Influencing Electronic Communication and Adherence to Best Practices

**DOI:** 10.2196/jmir.8.1.e2

**Published:** 2006-03-24

**Authors:** Robert G Brooks, Nir Menachemi

**Affiliations:** ^1^Florida State University College of MedicineTallahasseeFLUSA

**Keywords:** Email, electronic records, health information technology, electronic communication

## Abstract

**Background:**

With the public’s increased use of the Internet, the use of email as a means of communication between patients and physicians will likely increase. Yet, despite evidence of increased interest by patients, email use by physicians for clinical care has been slow.

**Objective:**

To examine the factors associated with physician-patient email, and report on the physicians’ adherence to recognized guidelines for email communication.

**Methods:**

Cross-sectional survey (March–May, 2005) of all primary care physicians (n = 10253), and a 25% stratified, random										sample of all ambulatory clinical specialists (n = 3954) in the state of Florida. Physicians were surveyed on email use with patients, adherence to recognized guidelines, and demographics.

**Results:**

The 4203 physicians	completed the questionnaire (a 28.2% participation rate). Of these, 689 (16.6%) had personally used email to communicate with patients. Only 120 (2.9%) used email with patients frequently. In univariate analysis, email use correlated with physician age (decreased use: age > 61; *P* = .014), race (decreased use: Asian background; *P* < .001), medical training (increased use: family medicine, *P* = .001; or surgical specialty, *P* = .007; but not internal medicine, *P* = .112), practice size (> 50 physicians, *P* < .001), and geographic location (urban 17.2% vs. rural, 7.9%; *P* < .001). Multivariate modeling showed that only practice size greater than 50 (OR = 1.94; 95% CI = 1.01-3.79) and Asian-American race (OR = 0.26; 95% CI = 0.14-0.49) were related to email use with patients. Remarkably, only 46 physicians (6.7%) adhered to at least half of the 13 selected guidelines for email communication.

**Conclusions:**

This large survey of physicians, practicing in ambulatory settings, shows only modest advances in the adoption of email communication, and little adherence to recognized guidelines for email correspondence. Further efforts are required to educate both patients and physicians on the advantages and limitations of email communication, and to remove fiscal and legal barriers to its adoption.

## Introduction

The Institute of Medicine’s vision for a high quality healthcare system includes the concept of patient-centeredness, which emphasizes the need to be responsive to patient preferences and needs [1]. Towards this goal, the use of email between physicians and patients is recognized as enhancing communication [2-4] and is generally favored by many patients [4,5]. Moreover, with the public’s increased use of the Internet, the use of email between physicians and patients will likely increase. Yet, physicians’ adoption of email to communicate with patients has been relatively slow with only modest increases in adoption rates in recent years [6,7].

The current literature on the subject of physician-patient email is generally focused on somewhat limited populations or attributes. Work has been done, for example, on the experiences of early physician [2,8] or patient [9] email users, physician attitudes [10] and concerns [11] towards using this communication medium and the general benefits [2,3,12] of doctor-patient email. Early work has identified appropriate content for physician-patient email and has highlighted the medico-legal issues associated with this practice [13-15]. Published work has also examined the nature and regularity of email inquires by patients [14] or their caregivers [16] with physicians [5]. Early studies have reported relatively low rates, generally between 6 [17] and 10 [5] percent, for physician-patient email. However, these previous statistics have typically been reported from surveys of patients and not necessarily of physician groups or practices. As a result, despite the increasing attention in the literature, few recent scholarly studies have comprehensively examined the frequency of physician-patient email use or the factors associated with this practice.

To help interested doctors benefit from email communication with patients, the American Medical Association (AMA) and the American Medical Informatics Association (AMIA) have adopted sets of guidelines for physicians [18,19]. It is unknown to what extent physicians comply with these best practice recommendations while emailing patients. The current paper specifically examines these issues directly by scientifically surveying a large sample of physicians in the state of Florida. In addition, it identifies numerous trends in mid-2005 that update previously identified developments in the use of physician-patient email.

## Methods

### Survery

As part of a statewide study of information technology (IT) use in the ambulatory setting, we surveyed 14921 physicians in Florida, using the State Department of Health’s list of allopathic and osteopathic physicians with clear and active medical licenses. The survey (see Multimedia Appendix 1) included a series of questions regarding the use of email from the office. In addition, those who personally use email to communicate with patients were asked to indicate which guidelines from a list, if any, they required their patients and staff to use. The list, which included 13 questions, represented items from the AMIA [18] and AMA [19] communication guidelines developed to specifically advise physicians on the use of patient email. 

The survey and a cover letter were sent in March, 2005, to all primary care physicians (general internists, pediatricians, family physicians, general practitioners and obstetricians/gynecologists) and a 25% stratified random sample of other specialists. Due to the nature of the study, we excluded those with a practice address outside of Florida and those who do not traditionally practice in the ambulatory setting (eg, radiologists, pathologists, anesthesiologists and emergency physicians). Each questionnaire was tracked by a six digit identifying code. After four weeks, nonrespondents were mailed a second cover letter and questionnaire to reiterate our interest in their participation. Those physicians who indicated, by phone or mail, that they were no longer actively treating patients (ie, retirement, or other reasons) were excluded. Surveys returned after the initial mailing because of unknown or changed address were remailed when an updated address was obtained. Completed questionnaires were returned by physicians via business-reply paid postage. Data were entered into a computer database and subjected to verification and cross-check methodologies. For example, the first batch of entered data by each staff member was 100% verified to prevent data entry errors. Subsequently, a minimum of 10% of all surveys were verified. If problems were encountered in a batch, they were fixed and the proportion verified was increased. If any patterns of data entry errors were detected in a batch, verification of the field for all surveys was made. The protocol was approved by the institutional review board at Florida State University.

### Statistical Analysis

The survey included demographic questions which enabled us to identify differences in the use of email by practice size, medical training, practice type, age, race, and gender. To examine practice size, we computed categories based on number of physicians practicing at a given location. Medical training (or "specialty") refers the area in which respondents said they spend the majority of their practice time in (ie, internal medicine, family medicine, pediatrics, and so on). Age was categorized by decade and included those less than 40 years, those aged 41-50, 51-60, and 61 or older.

To analyze the data, we first employed standard descriptive statistics and utilized chi-square analysis or Fisher’s exact test (as appropriate) to identify significant differences among the independent variables of interest. Next, we utilized binary logistic regression models to compute adjusted odds ratios. In these models, independent and covariate predictors included medical training (primary care or other), practice size and type as well as physicians’ age, race, and gender. Our dependent variable was email use with patients. In addition, using a similar model, we examined whether or not any of the predictors independently was related to adherence to the 13 communication guideline items described above. For this analysis, we collapsed all the medical specialties into primary care or other. Primary care was defined as family medicine, internal medicine, and pediatrics. All analyses were computed in SPSS version 13.0 and two-tailed significance was considered at the *P* < .05 level.

## Results

A total of 4203 returned surveys were available for the current study. This represents a 28.2% participation rate. Demographic and practice characteristics of the respondents are shown in Table 1. Overall, demographics of respondents did not differ from known characteristics of Florida physicians [20].

**Table 1:** Demographic and practice characteristics of responding physicians (n = 4203)

			**Results**	
Demographics of Respondents:				
	Age: Mean (range)		50.64	(30–86)
	Gender: Male		2479	(75.9%)
	Race/Ethnicity	
		Caucasian	2875	(68.4%)
		Hispanic	539	(12.8%)
		Asian	433	(10.3%)
		African-American	133	(3.2%)
		Other (or unknown)	223	(5.3%)

Practice Characteristics:				
	Mean years in current community		14.4	(< 1– 52)
	Mean years since medical school graduation (range)		21.4	(< 1– > 65)
	Specialty:^*^	
		Family Medicine	756	(18.3%)
		Internal Medicine	783	(18.9%)
		Pediatrics	602	(14.6%)
		Obstetrics/Gynecology	454	(11.0%)
		General Surgery	42	(1.0%)
		Surgical Specialty	393	(9.5%)
		Medical Specialty	709	(17.1%)
		Other^†^	397	(9.6%)
	Presence of an office computer		4015	(96.1%)
	Presence of Internet access		3812	(96.5%)
	High-speed access		2848	(85.3%)
	Dial-up connection only		404	(12.2%)

^*^Based on majority time spent in practice as reported by respondents.

^†^ Includes all other specialties, and physicians primarily in administrative roles.

### Physicians’ Use of Email With Patients

Overall, 689 physicians (16.6%) indicated that they personally used email from their office to communicate with patients. A majority of these doctors reported doing so rarely (314; 45.6%) or occasionally (255; 37%), with only 120 (17.4%) physicians saying they frequently used email to communicate with their patients (at least once on half of all business days). These 120 doctors represented 2.9% of 4148 physicians who responded to the email question in the survey. Physicians who frequently sent email to patients did not differ demographically from those who sent email only rarely or occasionally, except, of note, all 120 physicians who stated they frequently emailed patients practiced in urban areas (*P* = .048 compared to rural).

Using email to communicate with patients was first assessed by physician age, race, medical training, practice size, and to urban geographic practice location using univariate analysis (see Table 2). For example, physicians in the oldest age category (11.7% for those 61 years or older; *P* = .014) and those of Asian decent (7.2%) were least likely to engage in physician-patient email. Type of medical training also was related to email practices, in that family medicine doctors and surgical specialists were more likely to email patients than other groups. Although a significant difference was not noted between physicians who practice in single or multi-specialty practices, practice size itself was significantly related to the likelihood of email use. Groups of 50 or more physicians were significantly more likely (27.3%) to use email than those in smaller practices (14.5% to 22.7%; *P* < .001). Urban practice location was also significantly associated with physician-patient email use (17.2% vs. 7.9%; *P* < .001). Physicians who had high-speed Internet access (18.5% vs. 10.7%; *P* < .001), or indicated using an EHR system (25.4% vs. 13.9%; *P* < .001) were more likely to state that they sent email to patients.

When analyzed in a multivariate model, only two variables were noted to be statistically significant  predictors for email use. Physicians who practiced in groups of 50 or more were more likely than physicians in solo practice to communicate with patients via email (adjusted OR = 1.94; 95% CI = 1.01­–3.79). In addition, Asian-American respondents appeared to use email communication less commonly with patients then Caucasian physicians (adjusted OR = 0.26; 95% CI = 0.139–0.487).

**Table 2:** Number and percent of physicians who use email with patients in Florida (n = 689)

		**Number (percent) of physicians who use email with patients**		*P*-value^*^	**Adjusted Odds Ratios (95% CI)**	

**Total**		689	(16.6)			

**Age**						
	Less than 40 years old	79	(16.4)		1.00	
	41-50 years	197	(17.6)		1.09	(0.75–1.59)
	51-60 years	168	(18.2)		1.23	(0.83–1.81)
	61 years or older	56	(11.7)	.014	0.69	(0.42–1.12)
		
**Gender**						
	Male	410	(16.7)		1.00	
	Female	119	(15.3)	.34	0.87	(0.64–1.17)

**Race**						
	Caucasian non-Hispanic	522	(18.3)		1.00	
	African-American or Black	21	(16.0)		1.24	(0.66–2.34)
	Hispanic	78	(14.6)		0.82	(0.57–1.16)
	Asian	31	(7.2)		0.26	(0.14–0.49)
	Other race or unknown	37	(17.7)	< .001	0.91	(0.48–1.71)

**Specialty**						
	Family Medicine	154	(20.6)	.001^†^	^‡^see note below	
	Internal Medicine	114	(14.7)	.11		
	Pediatrics	86	(14.5)	.14		
	Obstetrics/Gynecology	75	(16.7)	.93		
	General Surgery	7	(16.7)	.98		
	Surgical Specialty	83	(21.4)	.007		
	Medical Specialty	113	(16.0)	.67		
	Other	46	(11.8)	.008		

**Practice type**						
	Single specialty	407	(15.2)		1.00	
	Multi specialty	81	(18.0)	.12	1.07	(0.73–1.58)

**Practice size**						
	Solo practice	176	(14.5)		1.00	
	2-9 physicians	330	(15.5)		1.01	(0.70 –1.32)
	10-49 physicians	87	(22.7)		1.11	(0.63–1.95)
	50 or more physicians	56	(27.3)	< .001	1.94	(1.01–3.79)

^*^ Univariate *P*-values, calculated by chi square, compare trends between groups.

^†^*P*-values for each specialty represent the comparison of the given specialty with all other groups.

^‡^ In multivariate analysis, we compared primary care physicians to other specialists; adjusted OR = 0.97 (0.77–1.24).

Of all physicians who did not currently use email with their patients, 13.4% indicated a future interest in doing so. An additional 52.8% expressed no desire to begin using email with patients and about one-third (33.8%) were undecided about future email use with patients.

### National Physician-Patient Email Guidelines

**Figure 1:**
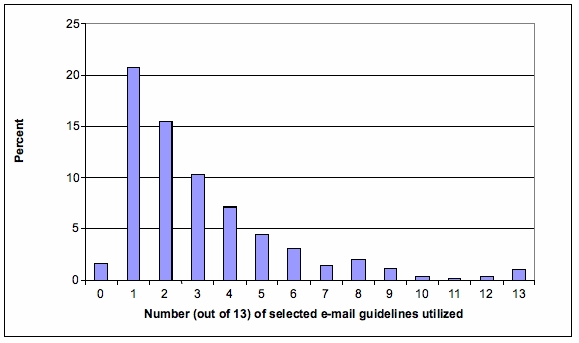
Number and percent of selected email guideline items being adhered to by physician practices in Florida (n = 689)

Of the 689 respondents who indicated using email with patients, only seven doctors (1.6%) indicated requiring their patients to abide by *all* the selected guideline items (see Figure 1).

Furthermore, only 46 physicians (6.7%) required their patients to comply with at least half (7) of the 13 guideline items (Table 3). The most common practice, among less than half of respondents, was printing the email communication and placing it in the patient’s chart (48%). The next most common practice, “informing patients about privacy issues with respect to email”, occurred among 36.3% of respondents. Adherence to any one of the other individual guideline items was infrequent, occurring in less than 25% of physician responses. Physician-respondents who stated that they frequently sent email to patients were more likely to adhere to 5 or more national guideline items (32.2% vs. 10.4%; *P* < .001). When analyzed by multivariate regression, physicians who were in primary care (adjusted OR = 1.95; 95% CI = 1.06–3.31) or in a practice of 50 or more physicians (adjusted OR = 8.07; 95% CI = 1.03–62.5) were more likely to follow 5 or more guideline items. Conversely, multivariate analysis of the group of physicians who followed less than 2 guideline items showed a significant negative correlation only with primary care (adjusted OR = 0.67; 95% CI = 0.48–0.98).

**Table 3:** Physicians’ self-reported adherence to recommended guideline items when emailing patients

**Nationally Recommended Policies**	**No. of Physicians(n = 689)**	**Percent**
Print email communication and place in-patients’ charts	331	48.0
Inform patients about privacy issues with respect to email	250	36.3
When email messages become too lengthy, notify patients to come in to discuss or call them	148	21.5
Establish a turnaround time for messages	111	16.1
Request patients to put their names or identification numbers in the body of the message	111	16.1
Send a new message to inform patient of completion of request	111	16.1
Establish types of transactions	110	16.0
Explain to patients that their message should be concise	70	10.2
Remind patients when they do not adhere to guidelines	55	8.0
Develop archival and retrieval mechanisms	57	8.3
Instruct patients to put category of transaction in subject line of message	48	7.0
Configure automatic reply to acknowledge receipt of patients’ messages	42	6.1
Request patients to use auto-reply features to acknowledge clinician’s message	28	4.1

### Nonpatient Email

Among the physician-respondents, 2593 (63%) indicated the use of email from their office for communication with groups other than patients. Most commonly, they reported the use of email to communicate with friends or family members (74.2%), other doctors (63.8%), and for business-related communications (50.1%). Less common (though still more common than email to patients) was email to hospitals (29.2%) and pharmaceutical companies (20.5%). Lastly, 12.9% of physicians suggested emailing some “other” group besides those listed above.

## Discussion

Patient-provider electronic mail has been previously defined as “computer-based communication between clinicians and patients within a contractual relationship in which the healthcare provider has taken on an explicit measure of responsibility for the client’s care” [19]. As such, it is an important tool for physician communication with patients in both general [21,22] and specialized [23,24] areas of medical practice. Despite the improved communication potential from the use of physician-patient email, the number of physicians electing to do so is still low, even though broadband Internet access is very common. Our finding of over 85 percent of physicians having high-speed Internet access is consistent with other US-based surveys [25].

Yet, the present study, conducted in mid-2005, found that only 16.6% of physicians in Florida used email with patients, and only 2.9% of the overall respondents used it frequently. This latter number, derived from physicians’ responses, suggests how rare email communication remains in clinical practice and is substantiated by studies showing the low number of patients who have ever sent email to a physician [5,26]. Although some patients do not yet have regular access to email [11], studies of the general public show both an increasing access to email accounts [27] and a general interest in email communication with their physicians [5,12]. From the perspective of the diffusion theory, physician-patient email is only now beginning to traverse the uphill slope of the adoption curve [28]. Yet, the fact that physicians are regularly using email from their offices to communicate with virtually all other entities (except patients), indicates that barriers seem to be specifically *impeding email use with patients*.

These barriers have been identified previously [2,15,29] and appear to be due to several specific fiscal and legal causes. Even though most email communications are asynchronous in nature, physicians spend valuable time and resources responding to email messages from patients [8,30,31]. This represents an “opportunity cost” to some physicians, particularly if the email system in place does not replace other modes of communication such as telephone messages, postal letters, etc [2]. In addition, the purchase and maintenance of encryption software, required to achieve maximum privacy, adds expense to the practice [32]. Only recently have several pilot programs in the United States begun to reimburse physicians for the expenses associated with direct email consultation [33-35].

The pace of email communication to patients has also been slowed by concerns from physicians [30] and staff [36] over general liability and privacy stemming from the recent Health Information Portability and Accountability Act [37-39]. For the interested reader, several excellent reviews exist that discuss the numerous legal and policy implications of physician-patient email and electronic health record use [40-43]. For those interested in the policy issues related to unsolicited email from patients, a seminal study by Eysenbach and Diepgen, which describes the policy implications, is recommended reading [44].

There may be a difference in perceptions between patients and physicians of the benefits accrued from the use of electronically available information. For example, a survey of patient use of the Internet for health information suggested that patients perceive more benefits and fewer risks than their physicians do, when this mode of information gathering is utilized [45]. A further study of these perceived differences in email benefits/risks is warranted.

Another important observation from the current study is that the use of email with patients occurs most frequently among certain groups of physicians. In one of the few studies that reported demographic information of physicians who do, and do not, regularly email patients, Gaster et al found that female physicians, younger physicians, and university-based clinic physicians were proportionately more likely to use patient email [10]. Community-based physicians, who more often offer primary care, tended to use email less than university- or county-hospital based clinics. In the present study, both family medicine physicians and surgical specialists were more likely to email patients. We believe the percentage of surgical specialists using email may be higher because they tend to work in larger practices (which were also more likely to use email). Family medicine doctors also have a higher likelihood of email use in Florida. We hypothesize this may be due to an ongoing health information technology educational program actively being pursued by the Florida and American Academies of Family Physicians, respectively. Similar to findings by Gaster et al, we found less email communication by older physicians. We believe this trend will disappear as the current physician workforce ages and younger physicians, with a higher general comfort level with information technology, appear in the workforce.

As email communication differs from traditional, written medical communication between physicians and patients and among providers, guidelines for best practices have been developed. These guidelines have emanated from both the medical [19,46-48] and health informatics [18] professions, as well as experts in the bioethics [49,50] and legal [51,52] fields. In the current study, we chose to design our survey questions around the guidelines found in two large US medical and informatics organizations because of their breadth and general availability [18,19]. The AMIA released its guidelines in 1998, and the AMA [18,19], in 2000. Both of these sets of recommendations are available online for physicians to review and utilize.

One of the most important findings of the current study is that few physicians were routinely utilizing these guidelines for email communication	with patients, despite their broad availability for several years. In this regard, the current study results are similar to those of Gaster and colleagues from a 2000-2001 survey of physician practices related to email use [10]. They found that 75% of physician-respondents never or rarely obtained consent to communicate with patients by email, 66% never or rarely discussed confidentiality or security concerns and 58% never or rarely documented email in the patient record. Importantly, a separate study by White et al found that the majority of patients involved with regular physician email communication do follow guidelines when they are educated about their nature and importance [14]. The findings by White et al, done from the patient’s perspective, coupled with the physician-oriented findings from our current study, suggest to us that the main barriers to guideline use may be more with the physician’s initiation than with the patient’s compliance.

The low rate of adherence to published physician-patient email guidelines may have several reasons. Among these reasons may be the lack of knowledge about the existence of guidelines by many practicing physicians; the lack of agreement with the guidelines (eg, not feeling that the guidelines are required in their particular practice), or an impracticality to their implementation. Unfortunately, the present study was not designed to determine reasons for not adhering to these recommended guidelines. However, given the results presented in the current study, the medical profession should consider further educating physicians about email communication, assess the barriers facing implementation, and better understand the practicality of utilizing the guidelines themselves.

We acknowledge that there are several important limitations of this study. First, we recognize that the survey response rate, although higher than comparable previous studies [22,53,54], may be a limitation. However, upon employing common methodologies used to detect bias, we failed to identify the presence of response bias. Second, as with other self-reported surveys, the study relies on the willingness and ability of participants to give accurate responses. Finally, because the purpose of the study was to identify the use of email by physicians in one state, the results of this study should be generalized to other geographic regions with caution.

To enhance email communication between physicians and patients, we believe that further work to educate both physicians and patients on the advantages and limitations of email correspondence is necessary. In addition, efforts are needed to deal with the fiscal barriers many physicians face in the regular use of email as a quality-enhancing tool in patient care. Although we are encouraged by recent efforts to reimburse physicians for email communication in several areas of the United States, most US physicians do not yet have access to these reimbursement programs. As these barriers are addressed in the United States, we believe email communication between physicians and patients will become better defined, better compensated and a resource for better clinical care of patients.

## Multimedia Appendix 1

Survey of Physician Information Technology use in Florida, developed by the Florida State University College of Medicine.
