# Supplementary material for: Physicians’ Use of Email With Patients: Factors Influencing Electronic Communication and Adherence to Best Practices
Source: J Med Internet Res. 2006 Mar 24;8(1):e2. doi: 10.2196/jmir.8.1.e2 (PMC1550692; doi:10.2196/jmir.8.1.e2)
Supplement: Supplementary file 1 [file jmir_v8i1e2_app1.pdf]

Use of Information Technology in Physician Practices

1. **Do you have access to a computer at your current office practice?**

   ☐ **YES** ☐ **NO -- PLEASE SKIP TO QUESTION #2**

   *If **YES**, please answer the following.*

   a. **Do you have internet access at your current office practice?**

      ☐ **YES** **What type of internet access do you have?** *(Please ✓ all that apply)*

      ☐ Dial-up ☐ High Speed (i.e. cable or DSL) ☐ Wireless

      ☐ **NO**

   b. **Do you <u>routinely</u> use the available computer? (at least once on ½ of all business days)**

      ☐ Yes ☐ No

   c. **Do other, non-physician, staff at your office use the available computer?**

      ☐ Yes ☐ No

   d. **Does the computer get used in the scope of your practice?**

      ☐ **YES** **For what type of functions?** *(Please ✓ all that apply)*

      ☐ Scheduling of patients appointments
      ☐ Billing/ charge capture
      ☐ Drug references/Medication interactions
      ☐ Access to reference materials
      ☐ Electronic prescribing of drugs
      ☐ Electronic order entry (e.g., labs, x-rays)
      ☐ Other: (*Please Specify)* ________________________________
      ☐ Patient registration
      ☐ Dictation
      ☐ Lab results
      ☐ Bills/claims submission
      ☐ Weight based dosing
      ☐ Patient records

      ☐ **NO**

2. **Do you currently <u>own</u> a personal digital assistant (PDA) (i.e., Palm Pilot or Pocket PC)?**

   ☐ **YES** ☐ **NO**

3. **Do you <u>routinely</u> use a PDA in your office practice? (at least once on ½ of all business days)**

   ☐ **YES** ☐ **NO**

   If Yes, for which of the following functions do you use your PDA? *(Please ✓ all that apply)*

   ☐ Drug references
   ☐ Medication interactions
   ☐ Access to reference materials
   ☐ Electronic Prescribing of medications
   ☐ Electronic order entry (e.g., labs, x-rays)
   ☐ Calendar and other organizer functions
   ☐ Other: (*Please Specify)* ________________________________
   ☐ Charge capture
   ☐ Patient records
   ☐ Lab results
   ☐ Bills/claims submission
   ☐ Weight based dosing
   ☐ Dictation

4. Do you <u>personally</u> use email from your office practice to communicate with <u>patients</u>?

☐ **YES** ☐ **NO -- PLEASE SKIP TO QUESTION #4C**

If YES, please answer the following:

a. How often do you email patients?

☐ Often (at least once on ½ of all business days) ☐ Occasionally ☐ Rarely

b. Which of the following policies, **<u>if any</u>**, do you require for e-mail with your patients?

- ☐ Establish a turnaround time for messages
- ☐ Inform patients about privacy issues with respect to e-mail
- ☐ Print e-mail communications and place in patient's chart
- ☐ Establish types of transactions (i.e., prescription refill, appointment scheduling, etc.)
- ☐ Instruct patients to put category of transaction in subject line of message
- ☐ Request patients put their name or identification number in body of message
- ☐ Configure automatic reply to acknowledge receipt of patient's message
- ☐ Send a new message to inform patient of completion of request
- ☐ Request patients use auto-reply feature to acknowledge reading clinician's message
- ☐ Develop archival and retrieval mechanisms
- ☐ Explain to patients that their message should be concise
- ☐ Remind patients when they do not adhere to guidelines
- ☐ When e-mail messages become too lengthy, notify patients to come in to discuss or call them

c. **If you <u>DON'T</u> personally use email with patients:** *Please answer the following:*

Would you like to communicate with your patients by email in the future?

☐ Yes ☐ No ☐ Don't Know Yet

5. Other than patients, do you use email from your practice with any other groups?

☐ **YES** ☐ **NO**

If YES, which of the following groups do you use email with? *(Please ✓ all that apply)*

- ☐ Family member or caregiver of patients
- ☐ Other doctors
- ☐ Business related communications (e.g., with insurers, pharmacies, etc.)
- ☐ Hospitals
- ☐ Pharmaceutical companies
- ☐ My personal friends or family members
- ☐ Other (please specify):____________________________

6. **Does your current office practice use a Registry or Disease Management software system?**

☐ **YES** ☐ **NO**

If **YES**, which of the following chronic diseases are followed? *(Please ✓ all that apply)*

- ☐ Diabetes
- ☐ Coronary Artery Disease
- ☐ Hypertension
- ☐ Heart Failure
- ☐ Preventive Care
- ☐ Other: (*Please Specify)*: ________________________________

7. **Does your current office practice have an Internet website available to patients?**

☐ **YES** ☐ **NO** If **NO**, do you plan to get a website? *(Please ✓ one)*

- ☐ **YES**, very soon (within 1 year)
- ☐ **YES**, but not within the next year
- ☐ **NO**

8. **Does your current office practice use electronic health records (EHR)?**

EHR is defined as a paperless form of the medical record that requires the provider to enter patient information (i.e., clinical notes) into a computer system instead of doing so on paper.

☐ **YES**

If yes, what YEAR best describes when you began using EHR in your practice ______________
(please indicate year)

If yes, please specify the vendor of your EHR system:__________________________________

☐ **NO** *Please answer the following:* **Are you considering getting EHR?** *(Please ✓ one)*

- ☐ Yes, very soon (within 1 year)
- ☐ Yes, but not within the next year
- ☐ No, I am not considering getting EHR at this time

9. **Do you personally routinely use Electronic Health records (EHR) in your office practice?**

☐ **YES** ☐ **NO**

If YES, Which of the following functions does your EHR include? *(Please ✓ all that apply)*

- ☐ Problem list
- ☐ Procedures
- ☐ Diagnoses
- ☐ Medication list
- ☐ Allergies
- ☐ Patient demographics (i.e., age, DOB, etc.)
- ☐ Clinical notes
- ☐ Electronic prescribing of medications
- ☐ Electronic order entry (i.e., labs or x-rays)
- ☐ Electronically available lab data/ results
- ☐ Electronically available x-ray results
- ☐ Electronic connection to pharmacy info
- ☐ Patient scheduling
- ☐ Weight-based dosing calculations
- ☐ Growth charting
- ☐ Clinical decision support
- ☐ Patient education materials
- ☐ Coding advice to physicians
- ☐ Advance directives
- ☐ Access to reference material
- ☐ Preventive service reminders
- ☐ Auto-updated insurance coverage info
- ☐ Offsite access/ log-in capability
- ☐ Other (*Specify)*:______________________

10. Please indicate how each potential barrier affects your decision to continue (or expand) using EHR. If you do not currently use EHR, please respond by indicating how much each barrier contributes to why you don't currently use EHR in your office practice.

	POTENTIAL BARRIERS			
**Productivity**	**Major Barrier**	**Minor Barrier**	**Not a Barrier**	**Not Applicable**
• Lack of time to acquire, implement such a system	☐	☐	☐	☐
• Entering data into computer can be cumbersome	☐	☐	☐	☐
• No time to learn how to use such a system	☐	☐	☐	☐
• The system would be difficult to use	☐	☐	☐	☐
• EHR may slow me down	☐	☐	☐	☐
• Disrupts workflow and/or office's physical layout to accommodate going to a computerized system	☐	☐	☐	☐
• Temporary loss of productivity and/or revenue during EHR system implementation phase	☐	☐	☐	☐
**Financial**				
• Inadequate Return on Investment (ROI)	☐	☐	☐	☐
• Upfront cost of hardware/software are too high	☐	☐	☐	☐
• Ongoing maintenance costs would be too high	☐	☐	☐	☐
**Technical**				
• Lack of uniform data standards within the industry	☐	☐	☐	☐
• Products available do not meet my needs	☐	☐	☐	☐
• Me and/or my staff don't have any technical knowledge	☐	☐	☐	☐
• Temporary loss of access to patient records if computer crashes or power fails	☐	☐	☐	☐
**Patients**				
• Privacy/confidentiality concerns (i.e., electronic records not secure)	☐	☐	☐	☐
• Patient resistance or not wanting their physicians to use EHR	☐	☐	☐	☐

11. How satisfied are you with the level of computerization in your current office practice?

☐ Very Satisfied ☐ Somewhat Satisfied ☐ Neutral ☐ Somewhat Dissatisfied ☐ Very Dissatisfied

12. Overall, how sophisticated of a computer user do you consider yourself?

☐ Very Sophisticated ☐ Sophisticated ☐ Neutral ☐ Unsophisticated ☐ Very Unsophisticated

13. Overall, how satisfied are you with your current medical practice?

☐ Very Satisfied ☐ Somewhat Satisfied ☐ Neutral ☐ Somewhat Dissatisfied ☐ Very Dissatisfied

DEMOGRAPHIC INFORMATION

14. Which of the following best describes the area in which you currently spend the majority of your practice time? (Please select only **one** choice)

- ☐ Family Medicine
- ☐ Internal Medicine
- ☐ Pediatrics
- ☐ OB/GYN
- ☐ General Surgery
- ☐ Surgical Specialty *(Specify)*______________________
- ☐ Medical Specialty *(Specify)*______________________
- ☐ Other *(Specify)* ______________________

15. Estimate the percent of your practice that is made up of patients in the following age groups:

0-18 years _____ %
19-44 years _____ %
45-64 years _____ %
65 years and over _____ %

16. Approximately what percentage of your patients have the following insurance coverage?

Medicare _____ %
Medicaid _____ %
Private insurance _____ %
Self-pay or uninsured _____ %

17. How many physicians, including yourself, work at the practice location where you spend the majority of your time?

___________ # of physicians

18. Which single setting best describes where the majority of your time is spent?

- ☐ Single specialty practice
- ☐ Multi specialty practice
- ☐ Hospital or Emerg. Dept. (hospital employee)
- ☐ Hospital-owned office-based practice (hospital employee)
- ☐ Group or staff model HMO
- ☐ Academic health center/ university setting
- ☐ Community health center
- ☐ County health department
- ☐ Other *(Specify)* _____________________

19. How long have you practiced . . .

In your current community? __________ **YEARS**
Total years in practice (since medical school graduation) __________ **YEARS**

20. Race/Ethnicity:

- ☐ White non-Hispanic
- ☐ African-American or Black non-Hispanic
- ☐ Hispanic
- ☐ Asian
- ☐ Other

Gender:

- ☐ Male
- ☐ Female

Age: __________________ (years)

21. If you are willing to participate in follow-up research related to this survey, please mark the following box: ☐ *(Your responses will always be kept confidential)*

☐ **Yes**, I would like to receive a summary of the findings. Email:___________________________

Thank you for your help!!!

Please return survey in the pre-addressed, postage-paid envelope to:
FSU Survey Research Laboratory Tallahassee, Florida 32306-2221
